# Characterization of a xylitol dehydrogenase from *Aspergillus flavus* and its application in l-xylulose production

**DOI:** 10.3389/fbioe.2022.1001726

**Published:** 2022-09-12

**Authors:** Anurag Kumar, Jinglin Li, Sanath Kondaveeti, Bakul Singh, Ramasamy Shanmugam, Vipin Chandra Kalia, In-Won Kim, Jung-Kul Lee

**Affiliations:** Division of Chemical Engineering, Konkuk University, Seoul, South Korea

**Keywords:** *Aspergillus flavus*, xylitol, dehydrogenase, thermostability, L-xylulose

## Abstract

An NAD^+^-dependent xylitol dehydrogenase from *A. flavus* (AfXDH) was cloned and successfully expressed in *Escherichia coli*. AfXDH gene sequence revealed an open reading frame of 1,110 bp, encoding a polypeptide of 369 amino acids with a calculated molecular mass of 38,893 Da. Among various polyols, sorbitol and xylitol were preferred substrates of AfXDH with K_m_ values of 16.2 and 16.9 mM, respectively. AfXDH showed the highest activity in Tris-glycine-NaOH buffer (pH 9.5) at 50°C; it required Zn^2+^ or Mn^2+^ for enzyme activity. The half-life at 40°C and half denaturation temperature (T_1/2_) was 200 min and 45°C, respectively. Bioinformatic analyses along with biochemical properties confirmed that AfXDH belonged to the medium-chain dehydrogenase/reductase family. AfXDH exhibits higher thermostability and *k*_*cat*_ values than those of other XDHs. The feasibility of using AfXDH in l-xylulose production was demonstrated. AfXDH, when coupled with *Streptococcus pyogenes* NADH oxidase, efficiently converted xylitol to l-xylulose with 97% yield, suggesting its usefulness for the industrial l-xylulose production from xylitol.

## Introduction

Rare sugars exist in small amounts as natural products and hold vast potential for industrial applications. These sugars are increasingly important in the food industry and in the development of new therapeutic compounds since rare sugars are effective in controlling diabetes (e.g., low-calorie sweeteners) and are also used as building blocks for antiviral and anticancer drugs (Gumina et al., 2007; Espinosa and Fogelfeld, 2010; Beerens et al., 2012). Owing to these remarkable capabilities, rare sugars are of significant interest to researchers. d- or l-xylulose, a ketopentose, is scarce and is therefore categorized as a rare sugar. In particular, l-xylulose is an important substrate in the pentose phosphate pathway. Phosphorylation of xylulose by xylulokinase generates xylulose-5-phosphate, which is the key intermediate in all living organisms (Chiang et al., 1981a). Furthermore, ethanol is produced by the fermentation of l-xylulose using yeast (Chiang et al., 1981b). l-Xylulose has several applications, including its use as a potential inhibitor of various glucosidases (Leang et al., 2003) and in medicine. For example, it is a reliable indicator of acute or chronic hepatitis (Granström et al., 2005). It can also be used as a starting material to produce other important rare sugars, such as l-xylose, which functions as an antiviral and antitumor agent (Ma et al., 1997; Lim et al., 2002; Hofmann et al., 2005; Chevrier et al., 2006). Consequently, to better understand the application of l-xylulose, a crucial step is to produce this sugar in large quantities for use as an initial substrate in various reactions.

On the basis of amino acid sequence, molecular mass, and cofactor-binding site, the dehydrogenases/reductases are classified into short-chain dehydrogenases/reductases (SDRs), medium-chain dehydrogenases/reductases (MDRs), and long-chain dehydrogenases/reductases (El-Kabbani et al., 2004). Most XDHs belong to the MDRs superfamily and catalyze NAD(P)^+^-dependent oxidation of xylitol to l-xylulose (Negm and Loescher, 1979; El-Kabbani et al., 2004). In filamentous fungi, however, l-xylulose reductases catalyze the NAD(P)H-dependent reduction of l-xylulose to xylitol in l-arabinose and glucuronic acid catabolism and belong to the SDR superfamily (Metz et al., 2013). Xylose reductase and XDH are responsible for the assimilation of xylose into eukaryotic metabolism (Doten and Mortlock, 1985; Karhumaa et al., 2007). Xylose reductase reduces xylose to xylitol and XDH oxidizes xylitol to xylulose. Additionally, XDH is necessary for the efficient fermentation of pentose sugars contained in agricultural byproducts to produce ethanol. Although several XDHs have been reported, their catalytic efficiency and stability were not adequate for the biocatalytic production of l-rare sugars from their corresponding substrates.

Nicotinamide adenine dinucleotide (NADH) oxidases catalyze NADH oxidation by simultaneously reducing oxygen (O_2_) to hydrogen peroxide (H_2_O_2_) or to water (Riebel et al., 2003). NADH oxidases belong to the group 3 flavoprotein disulfide reductase superfamily and contain a cysteine sulfenic acid residue. NADH oxidases are effective in the regeneration of oxidized cofactor (NAD^+^). Since most proteins are inactivated upon exposure to H_2_O_2_ and no harmful secondary products are produced in the coupled cofactor regeneration reaction, water-forming NADH oxidases, in particular, have received more attention than H_2_O_2_-forming NADH oxidases (Gao et al., 2012).

In the present study, we report a highly efficient novel XDH from *A. flavus* (AfXDH). Based on its biochemical characteristics and amino acid sequence, AfXDH is classified as a member of the MDR superfamily with two metal binding sites. We also demonstrate the application of AfXDH in l-xylulose production from xylitol by coupling it with NADH oxidase from *S. pyogenes* (SpNOX).

## Materials and methods

### Materials, bacterial strains and culture conditions

The detailed source of materials and chemicals were given in the Supplementary Material. *E. coli* DH5α and *E. coli* BL21 (DE3) strains were purchased from Thermo Fisher Scientific (MA, United States) and New England Biolabs (MA, United States), respectively, and used as hosts for plasmid transformation and expression. The *A. flavus* NRRL3357 strain was obtained from the ARS Culture Collection (NRRL). *A. flavus* NRRL3357 was grown at 28°C in YM broth containing yeast extract 3 g L^−1^, malt extract 3 g L^−1^, and peptone 5 g L^−1^, and dextrose 10 g L^−1^ with shaking at 200 rpm. *E. coli* DH5α and *E. coli* BL21 (DE3) strains were used for plasmid transformation and expression, respectively.

### Amplification of *xdh* gene from *A. flavus* NRRL3357

The *A. flavus* NRRL3357 genomic sequence and XDH protein sequence were accessed from the NCBI site (www.ncbi.nlm.nih.gov). *A. flavus* NRRL3357 was cultured and genomic DNA was obtained using the DNA Purification Kit (Promega). The *xdh* gene was amplified from *A. flavus* NRRL3357 genomic DNA using the two primers, 5′–GGG​ATC​CAT​GGC​TAC​AGA​TAC​TCA​TCC​C–3′ and 5′–GAG​CTC​CTA​TAC​TGA​AGC​TTG​CTT​TGC–3′, by polymerase chain reaction (PCR).

### Cloning and expression of the *xdh* and nox genes

The amplified *xdh* gene with restriction sites *BamH*I and *Sac*I was cloned into pGEM-T vector and transformed into the *E. coli* DH5α strain. Plasmid DNA and the expression vector pQE80L were digested with *BamH*I and *Sac*I to release the *xdh* gene and create a nick in the pQE80L, respectively. The *xdh* gene was released from the pGEM-T vector and was ligated with the pQE80L to generate a recombinant plasmid, pQE80L-*xdh*. The pQE80L-*xdh* expressed XDH with a 6xHis tag. The pQE80L-*xdh* was transformed into *E. coli* BL21 (DE3) and the XDH was expressed using isopropyl-β-d-thiogalactopyranoside (IPTG, 0.5 mM) at 25°C. The induced cells were then harvested at 4°C by centrifugation for 20 min at 4,000 × *g* and rinsed with lysis buffer (pH 8.0, 50 mM NaH_2_PO_4_, 10 mM imidazole, 300 mM NaCl). The *spnox* gene from *S. pyogenes* was cloned into pET28a, expressed in *E. coli* BL21 (DE3), and purified as described previously (Gao et al., 2012; Li et al., 2018). Detailed expression and purification results of SpNOX were given in the (Supplementary Figure S1).

### Purification of *A. flavus* and enzyme assay

The recombinant 6xHis-tagged AfXDH was purified as described previously for other dehydrogenases (Tiwari et al., 2010). The activity of AfXDH was assayed by monitoring the change in A_340_ upon oxidation of NADH at room temperature. The assay mixture contained 2 mM NAD^+^, 200 mM of xylitol, and AfXDH in 20 mM Tris-glycine buffer (pH 9.5). The reaction was initiated by adding xylitol. Enzyme assays were performed in triplicate using the purified AfXDH.

### Optimum pH and temperature

The optimum pH of the recombinant AfXDH was determined using 20 mM sodium phosphate (pH 6.0–7.0), 20 mM Tris-HCl (pH 7.5–8.5), and 20 mM Tris-glycine (pH 9.0–10.0) buffers. The optimum temperature for the AfXDH activity was determined at different temperatures ranging from 20 to 60°C. The enzyme stability was assayed by incubating the purified AfXDH in 20 mM Tris-glycine buffer (pH 9.5) containing 0.1 mM Zn^2+^. At specific timepoints, samples were withdrawn, and remaining activity was determined.

### Substrate specificity

The substrate specificity of the purified AfXDH was determined under optimum conditions using various substrates such as sorbitol, xylitol, erythritol, glycerol, ribitol (adonitol), mannitol, galactitol, maltitol and d/l-arabinitol (200 mM).

### Effect of metal ions

The purified AfXDH was dialyzed at 4°C against 20 mM Tris-glycine buffer (pH 9.5) containing 10 mM ethylenediamine-tetraacetic acid (EDTA) for 12 h, and was dialyzed against 20 mM Tris-glycine-NaOH (pH 9.5). Then, the dialyzed enzyme was assayed using 200 mM xylitol in the presence of MnCl_2_, CoCl_2_, CaCl_2_, HgCl_2_, ZnCl_2_, MgCl_2_, BaCl_2_, CuSO_4_, and KCl at concentrations ranging from 0.05 to 5 mM.

### Kinetic parameters

The kinetic parameters of AfXDH were determined from triplicate measurements in 20 mM Tris-glycine buffer (pH 9.5), 0.1 mM Zn^2+^, 5–300 mM xylitol, and 0.1–3 mM NAD^+^. The *K*_*m*_ and V_max_ values were determined *via* the Michaelis–Menten equation using Prism 5 (GraphPad, Inc., CA, United States).

### High-performance liquid chromatography-evaporative light scattering detector analysis

To evaluate the reaction products obtained after the conversion of sorbitol and xylitol, we analyzed the reaction products from *in vitro* reactions using purified AfXDH by high-performance liquid chromatography (HPLC) (UltiMate 3000, Dionex, Idstein, Germany). A Shodex Sugar SP column (8.0 μm × 300 mm, Showa Denko, Tokyo, Japan) was used and maintained at 85°C. The eluent was H_2_O at a flow rate of 1.0 ml min^−1^. Peaks were detected using an evaporative light scattering detector (ELSD) detector.

### Homology modeling and substrate docking

The homology models were generated using the Prime module in Schrödinger suite 2019 (Schrondinger, NY, United States) (Schrödinger Release 2020-2, 2020). The multiple sequence alignment of XDH sequences was performed using Schrödinger Maestro (Schrödinger, NY, 2020). For comparative analysis, structure information was obtained from the Protein Data Bank (PBD) (Berman et al., 2000). The modeled structures were further analyzed by SAVES server (Koivistoinen et al., 2012) for Ramachandran plot. The docking studies were performed by Extra Precision (XP) glide (Bowers et al., 2006) in Schrödinger suite 2019.

### Molecular dynamics simulation

Molecular dynamics of AfXDH and ReXDH in complex with xylitol was performed by Desmond (Harder et al., 2016) Molecular Dynamics package of Schrodinger 2019. The OPLS3 (Jorgensen et al., 1996) force field was used both for protein atoms and xylitol to generate topologies for molecular dynamics (MD) simulation. All the systems were solvated in orthorhombic box and neutralized by Na^+^ ions as system set up method. After system was built, each system was undergone into 8 stages of MD simulation inclusive of minimization, equilibration, and final step of production run. All the production runs were carried out for 50 ns for each system, AfXDH and ReXDH. The snapshots were saved at regular interval of 1 ps.

## Results and discussion

### Cloning and heterologous expression of the *xdh* gene from *A. flavus* NRRL3357

**FIGURE 1:**
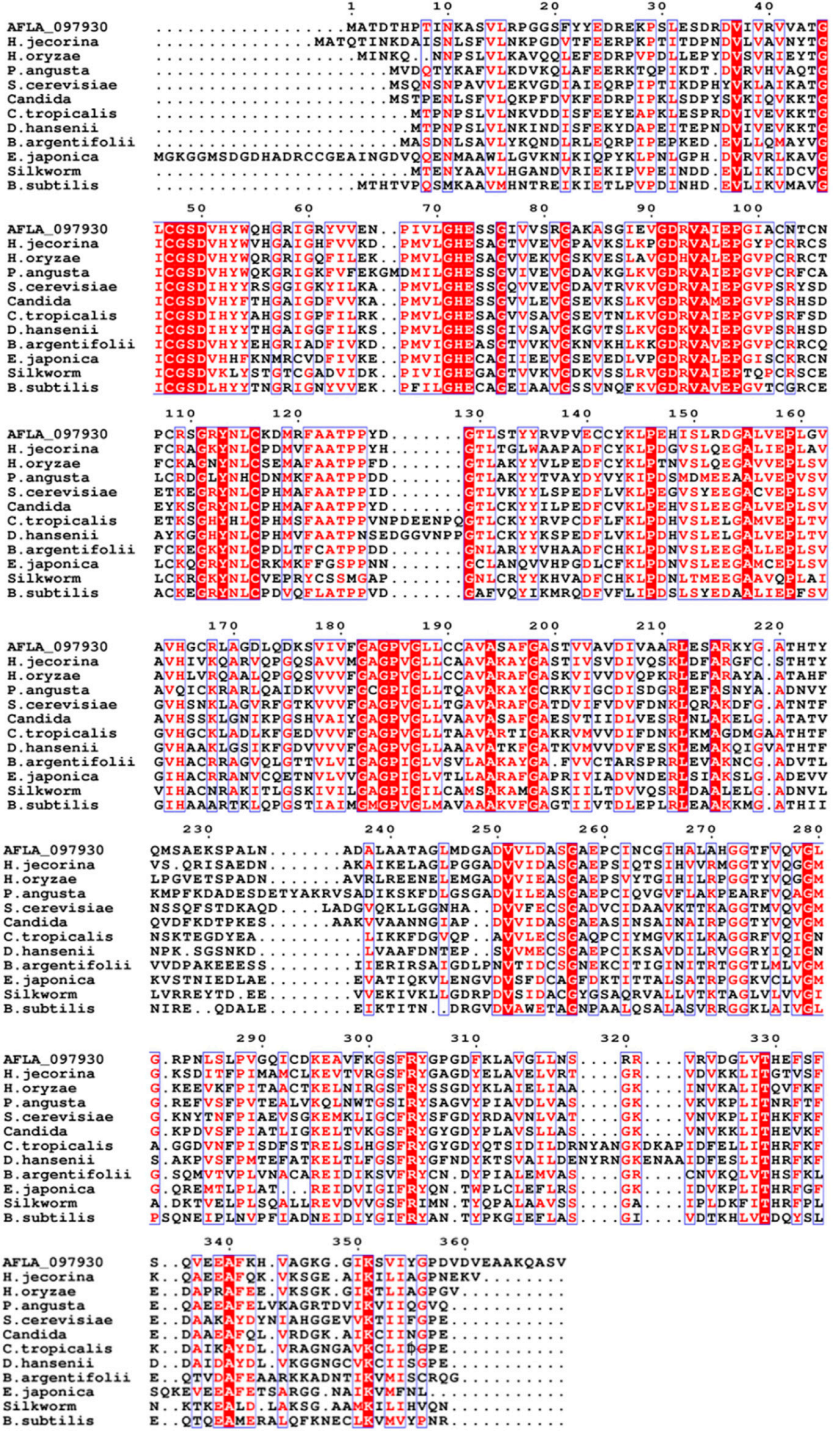
Multiple sequence alignment of *A. flavus* XDH with selected medium-chain reductase/dehydrogenases (MDRs). AFLA_097930, *Aspergillus flavus* XDH; *H. jecorina*, *Hypocrea jecorina* MDR; *H. oryzaea*, *Hirschmanniella oryzae* MDR; *P. angusta*, Pichia angusta MDR; *S. cerevisiae*, *Saccharomyces cerevisiae* MDR; Candida, *Candida sp.* MDR; *C. tropicalis*, *Candida tropicalis* MDR; *D. hansenii*, *Debaryomyces hansenii* MDR; *B. argentifolii*, *Bemisia argentifolii* MDR; *E. japonica*, *Eriobotrya japonica* MDR; Silkworm, *Bombyx mori* MDR; *B. subtilis*, *Bacillus subtilis* MDR. The red column shows conserved residues in the alignment, the dot (.) represents the gap, and the black sequences show non-conserved residues.

**FIGURE 2:**
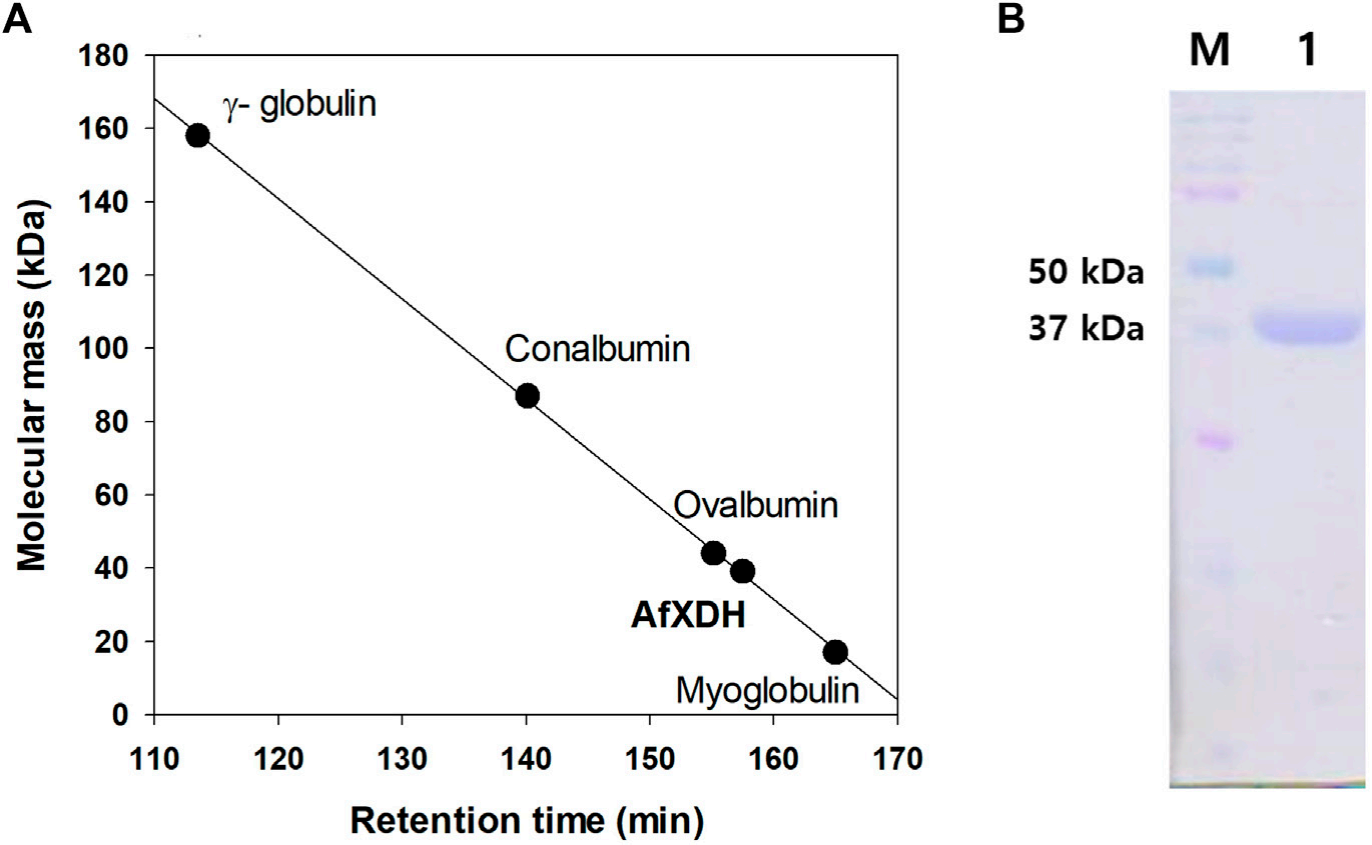
Determination of the molecular mass of *A. flavus* XDH by gel filtration chromatography and SDS–PAGE. **(A)** Gel filtration chromatography of AfXDH. To determine the molecular mass of AfXDH, the column was calibrated with *γ-*globulin (MW 158,000), conalbumin (MW 87,000), ovalbumin (MW 44,000), and myoglobin (MW 17,000) as reference proteins (GE Healthcare, United Kingdom). **(B)** SDS–PAGE of AfXDH. Lane M, Molecular mass Marker; Lane 1, purified recombinant AfXDH.

The *xdh* gene encoding XDH from *A. flavus* (GenBank accession number: XM_00238625) was 1,110 nucleotides long with an open reading frame of 369 amino acids. It possessed the NAD-binding Rossmann fold domain [(GXGPXG)] (position 182–187; where X is any amino acid), the catalytic Zn^2+^-binding site (Cys47, His72, Glu73, and Glu158), and the structural Zn^2+^-binding site (Cys102, Cys105, Cys108, and Cys116) highly conserved among MDRs (Figure 1). According to these bioinformatics studies, we considered AfXDH to be an XDH (a member of MDR) with two metal binding sites. The *Afxdh* gene was cloned into the pQE80L plasmid between the *Sac*I and *BamH*I sites and expressed in *E. coli* BL21 (DE3) with a 6xHis affinity tag at C-terminal. Gel filtration chromatography of the purified 6xHis-tagged AfXDH protein showed a symmetrical peak between myoglobin and ovalbumin, corresponding to a Mr of 39 kDa (Figure 2A). SDS-PAGE of the purified AfXDH exhibited a single band at ∼39 kDa (Figure 2B), suggesting that the native AfXDH is a monomer.

### Optimum pH and temperature

**FIGURE 3:**
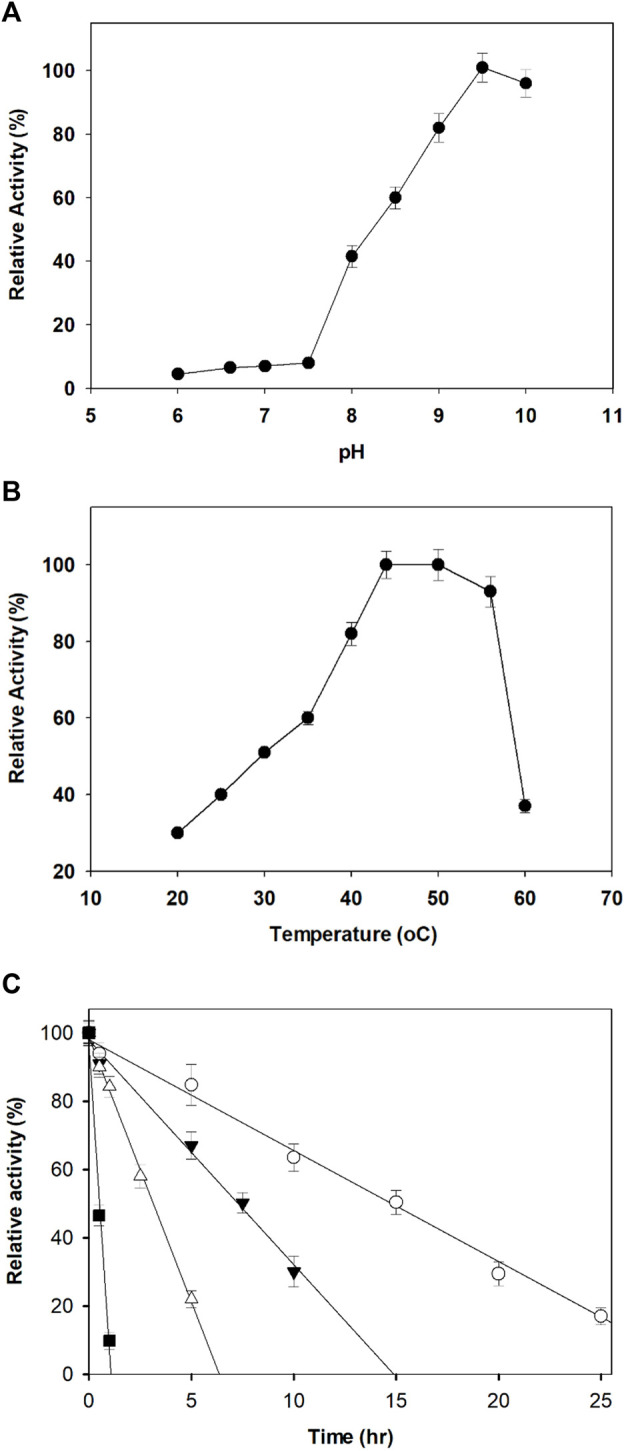
Effect of pH **(A)** and temperature **(B)** on the activity of AfXDH. Enzyme assays were performed under standard conditions in the presence of 200 mM xylitol. Activities at the optimal temperature and pH were defined as 100%. Three different buffers were used; sodium phosphate (20 mM) (pH 6.0–7.0), 20 mM Tris-HCl (pH 7.5–8.5), and 20 mM Tris-glycine-NaOH (pH 9.0–10.0). The purified enzyme exhibited temperature and pH optima at 50°C and 9.5, respectively. **(C)** Thermal stability of the purified AfXDH. Recombinant AfXDH was incubated at various temperatures, ranging from 30 to 55°C. After incubation for 10 min, the enzyme samples were chilled on ice; subsequently, their residual activity was determined by observing the relative activity of AfXDH at 30°C (open circle), 40°C (filled triangle), 50°C (open triangle), and 55°C (filled square). Each value represents the mean of triplicate measurements and varied from the mean by not more than 15%.

The optimum pH for the dehydrogenation of xylitol by purified AfXDH was 9.5 with 43% (28.4 U/mg-protein), 60% (39.6 U/mg-protein), 83% (54.8 U/mg-protein), and 92% (60.7 U/mg-protein) maximum activity at pH 8, 8.5, 9, and 10, respectively (Figure 3A). Maximal AfXDH activity at an alkaline pH optimum is a common feature of similar dehydrogenases/reductases obtained from various microorganisms. The optimum dehydrogenation temperature was found to be 50°C (Figure 3B). The AfXDH retained more than 95% of the maximum activity when analyzed at 55°C. In the presence of Zn^2+^, the half-life (t_1/2_) values of AfXDH were 15, 7.4, and 3.2 h at 30, 40 and 50°C, respectively. However, the AfXDH stability decreased significantly at 55°C with a t_1/2_ value of 30 min (Figure 3C). The t_1/2_ values of AfXDH (200 and 120 min) at 40 and 50°C are the longest t_1/2_ values among all characterized XDHs until now. The half denaturation temperature (T_1/2_) of AfXDH was determined to be 45°C by analyzing the remaining activities after heat treatment at various temperatures varying from 25 to 55°C.

### Substrate and coenzyme specificity

Xylitol, sorbitol, ribitol, galactitol, mannitol, meso-erythritol, glycerol, maltitol, and d/l-arabinitol (200 mM) were used as substrates to estimate the substrate specificity of AfXDH. The enzyme had a high preference for xylitol followed by sorbitol and showed low activity with other polyols. The coenzyme specificity of AfXDH was also examined; it exhibited coenzyme preference for NAD(H) over NADP(H). With xylitol as the substrate, the activity with NADP^+^ was 0.7% of the activity observed with NAD^+^, when 1.5 mM NAD^+^ or NADP^+^ was used as the coenzyme. AfXDH was exclusively an NAD^+^-dependent enzyme showing almost no activity with NADP^+^.

### Effects of metal ions and ICP-MS analysis

The activity of the AfXDH (1 mM) was thoroughly inhibited by Hg^2+^ and Cu^2+^ and strongly inhibited by Co^2+^ and K^+^. Slight inhibition was observed in the presence of Mg^2+^, Ba^2+^, and Co^2+^. AfXDH activity was stimulated by Mn^2+^. However, the highest activity was obtained using Zn^2+^ (0.1 mM), which enhanced AfXDH activity by 2.73-fold compared to the control in the absence of supplementary metal ions. The AfXDH activity was found to be dependent on divalent metal ions (Table 1).

**TABLE 1:** Effect of different metal ions on the activity of AfXDH. The purified enzyme was assayed in the standard assay condition with 0.1 or 5 mM metal ions. The activity of AfXDH measured in the absence of metal ions was set as 100%. Each value represents the mean of triplicate measurements and varied from the mean by not more than 15%.

Metal ions	Relative activity (%) 0.1 mM	Relative activity (%) 5 mM
None	100	100
Zn^2+^	273 ± 3	23.1 ± 5.0
Mg^2+^	61.4 ± 4.1	96.5 ± 6.4
Mn^2+^	162 ± 6	109 ± 8
Ca^2+^	71.8 ± 7.0	107 ± 8
Fe^2+^	24.3 ± 4.8	0
Cu^2+^	0	0
Co^2+^	51.7 ± 2.3	56.8 ± 4.3
Hg^2+^	0	0
Ba^2+^	115 ± 3	152 ± 6
K^+^	62.4 ± 7.2	0

ICP-MS analysis was performed to analyze the presence of metals in the purified AfXDH, we subjected the recombinant protein to. On the basis of the molecular mass of 38.9 kDa, 1 μmol of the AfXDH was found to contain 2 μmol of Zn^2+^. The sequence alignment (Figure 1) and ICP-MS analysis suggest that AfXDH contains two zinc-binding sites per monomer of AfXDH: a catalytic Zn^2+^ and a second structural Zn^2+^. According to multiple sequence alignment analysis of AfXDH with other XDHs, catalytic Zn^2+^ can bind to CYS47, HIS72, GLU73, and GLU158 (Koivistoinen et al., 2012). In addition, AfXDH activity was completely lost upon treatment with 10 mM EDTA and restored by the addition of 0.1 mM Mn^2+^ or Zn^2+^.

### Kinetic parameters of *A. flavus*

**FIGURE 4:**
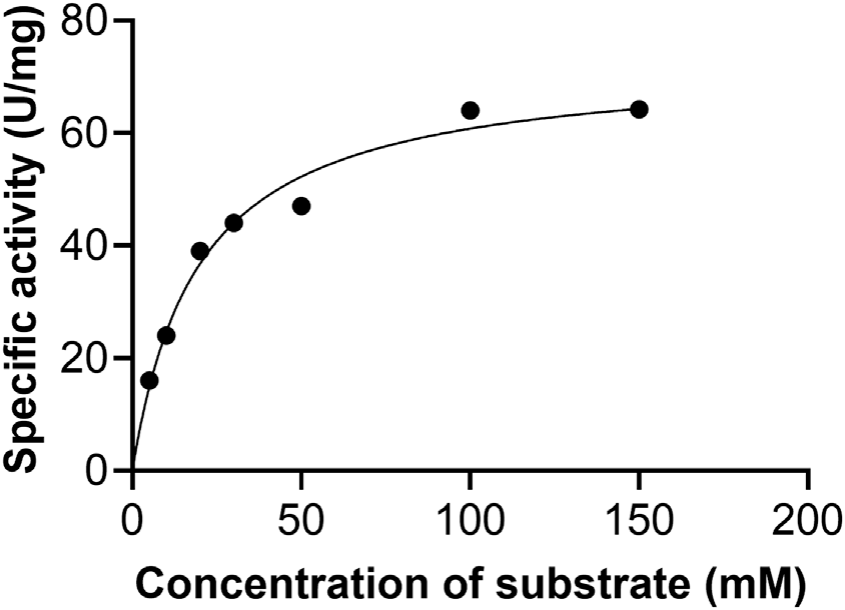
Effect of xylitol concentration on the activity of AfXDH. XDH activity of the enzyme was measured in the presence of indicated concentrations of the substrate at pH 9.5. The data represents an average of all statistically relevant results with a standard deviation of less than 15%.

Initial velocities were determined at pH 9.5 in the standard assay mixture. The kinetic parameters for AfXDH activity were analyzed using substrate concentrations varying from 5 to 300 mM (Figure 4). Maximum AfXDH activity was observed with a substrate concentration of 150 mM under the assay conditions. The *K*_m_, _xylitol_ value of AfXDH was found to be 16.2 mM (Table 2), which was lower than those of other XDHs from *Fusarium oxysporum* (Panagiotou et al., 2002) and *Pichia stipites* (Watanabe et al., 2005) but higher than XDHs from *Pachysolen tanophilus* (Morimoto et al., 1986) *and Bacillus pallidus* (Takata et al., 2010). The *k*_cat_ */K*_m_ for xylitol was 2.88 s^−1^ mM^−1^. When the NAD^+^ concentration increased from 0.1 to 3 mM, Michaelis–Menten type of kinetics were observed for AfXDH activity. The *K*_m_, _NAD+_ value was 0.15 mM. The properties of various XDHs have been compared in Table 2. AfXDH shared similar characteristics with other reported XDHs, such as M_r_ (∼39 kDa), and had high affinity toward NAD^+^. However, notably, AfXDH had the highest *k*_cat_ value (48.7 s^−1^) and thermostability (t_1/2_ = 200 min at 50°C) among all previously characterized XDHs (Tiwari et al., 2010; Tiwari et al., 2012).

**TABLE 2:** Biochemical and kinetic properties of xylitol dehydrogenases from various organisms.

Organism	Subunit M_r_ (kDa)	Optimum temperature	Metal ion	*K*_*m*_		k_cat_ (xylitol)	k_cat_ */K*_*m*_ (xylitol)	Thermal stability (t_1/2_)	References
				Coenzyme (NAD^+^)	Substrate (xylitol)				
*Fusarium oxysporum*	48	45	Mn^2+^	0.14	94	NR	NR	<60 min	Panagiotou et al. (2002)
*Bacillus pallidus*	28	55	NR	0.034	2.06	2.74	1.33	<10 min	Takata et al. (2010)
*Pachysolen tannophilus*	40	55	K	NR	11	NR	NR	<5 min	Morimoto et al. (1986)
*Pichia stipitiswt*	39	35	Mg^2+^	0.38	21.7	17.36	0.8	NR	Watanabe et al. (2005)
*Rhizobium etli CFN42*	34	70	Mg^2+^	0.25	12.1	3.025	0.25	120 min	Tiwari et al. (2010)
*Aspergillus flavus*	39	50	Zn^2+^	0.17	16.9	48.67	2.88	200 min	This study

NR, not reported.

### Homology modeling and docking analysis of XDHs

The multiple sequence alignment of AfXDH, *Rhizobium etli* XDH (ReXDH), *Bemisia argentifolli* XDH (PDB ID:1E3J), and *Homo sapiens* XDH (PDB ID: 1PL6) is shown in Supplementary Figure S2 (Banfield et al., 2001; Pauly et al., 2003). AfXDH was found to have sequence identities of 45%, 43.1%, and 38.0% with 1PL6, 1E3J, and ReXDH, respectively. The highly conserved active site residues (SER, GLU, and ARG) of XDHs have been reported (Lunzer et al., 1998; Filling et al., 2002; Ehrensberger et al., 2006). The active site residues, SER49, GLU158, and ARG304 of AfXDH correspond to SER42, GLU152, and ARG293 of ReXDH, and well conserved in all the XDHs. The coordinating residues (CYS44, HIS69, and GLU70) with catalytic zinc were also completely conserved (Supplementary Figure S2). The homology model of AfXDH (Supplementary Figure S3) obtained using IPL6 and 1E3J as templates was further validated by Ramachandran plot (Supplementary Figure S4) with 92.1% of residues in favored region, 6.2% in allowed regions, and 1.7% in disallowed regions. AfXDH has higher catalytic activity towards xylitol than other XDHs including ReXDH shown in Table 2. Homology model of ReXDH was generated using the same protocol used for AfXDH modeling. The final model structure of ReXDH was validated by Ramachandran plot (Supplementary Figure S5) where 86.9% of residues in favored regions, 5.6% in allowed regions, and 7.6% in disallowed regions.

**FIGURE 5:**
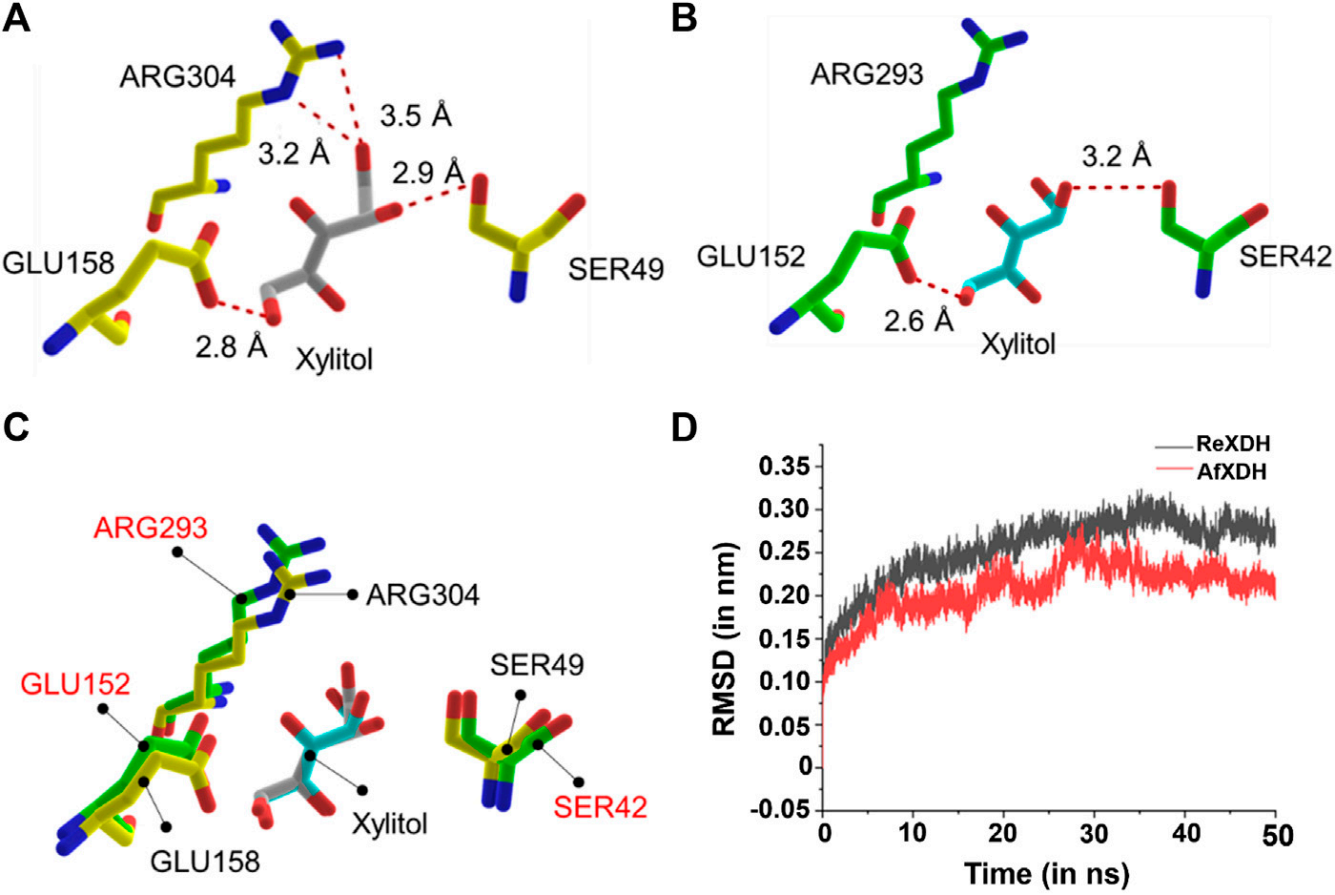
The docked complex of **(A)** AfXDH and **(B)** ReXDH with xylitol bound as substrate. The red dashed lines represent hydrogen bonds with donor-acceptor atom pair distance (in Å). Yellow stick model for AfXDH and green stick for ReXDH complex. **(C)** The superimposed active site residues of AfXDH (black label) and ReXDH (red label). **(D)** RMSD (in nm) analysis of AfXDH (red line) and ReXDH (black line) for 50 ns MD simulation.

To gain insight into the higher enzyme activity of AfXDH than other XDHs, we docked xylitol into both AfXDH and ReXDH. The docking energy of xylitol obtained for AfXDH and ReXDH from Glide XP method was found to be −6.077 (kcal/mol) and −4.857 (kcal/mol), respectively. The active site residues of AfXDH (SER49, GLU158, and ARG304) formed four hydrogen bonds with xylitol (Figure 5A). However, in the case of ReXDH, only two hydrogen bonds were formed between xylitol and GLU152 and SER42 (Figure 5B). The active site residues conserved in both the XDHs have similar structural fold in binding the substrate (Figure 5C). The greater number of hydrogen bonds formed by AfXDH with xylitol as compared to ReXDH is likely to contribute to its higher activity. The distances between donor and acceptor atoms in hydrogen bond for AfXDH and ReXDH are given in Supplementary Table S1.

### Molecular dynamics of *A. flavus* and *Rhizobium etli* XDH

The root-mean-square deviation (rmsd) of backbone atoms of AfXDH and ReXDH in complex with xylitol is shown (Figure 5D). The backbone atoms of AfXDH initially started to shoot up from 0.00 to 0.20 nm during 0–10 ns time. After 10 ns, the structure is found to be stable with no fluctuations at all in backbone atoms till 16 ns with rmsd in the range of 0.18–0.19 nm. AfXDH showed highest fluctuation at 28 ns with rmsd of 0.28 nm. In subsequent time, the backbone atoms stabilized more and maintained the rmsd of 0.20 nm until the end of the simulation. In case of ReXDH, the stability of backbone atoms was found to be less throughout the simulation with highest fluctuation in rmsd of 0.32 nm at 35 ns and overall rmsd in the range of 0.25–0.30 nm. The rmsd analysis clearly signifies the binding of xylitol in the active site of AfXDH is stable than ReXDH.

### Conversion of xylitol to l-xylulose by *A. flavus* coupling with *S. pyogenes*

**FIGURE 6:**
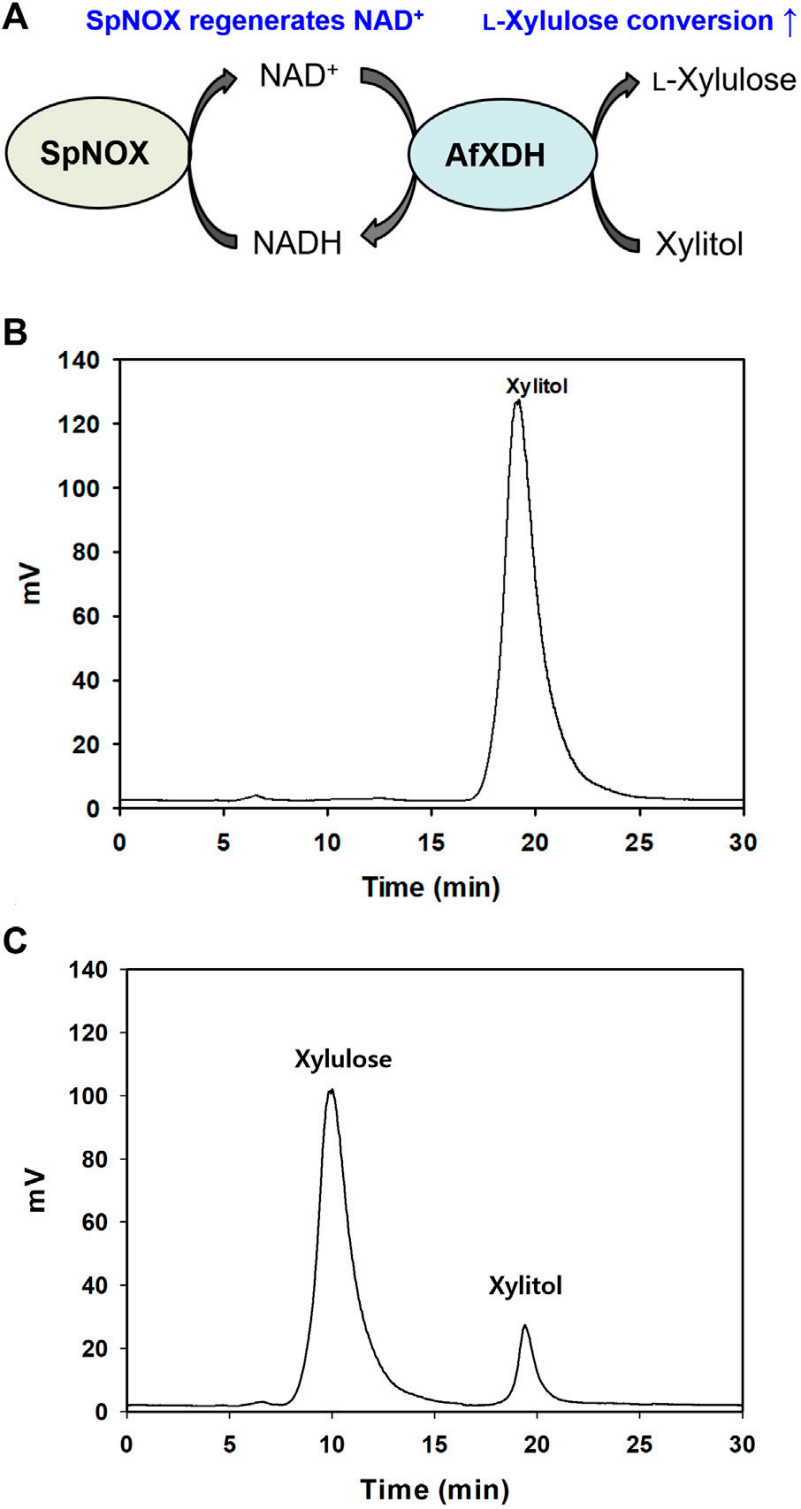
**(A)** Schematic diagram of l-xylulose production coupling with SpNOX. **(B)** Xylulose production without SpNOX, **(C)** Xylulose production coupling with SpNOX. The reaction continued for 12 h.

There is considerable interest in studies related to polyol dehydrogenases owing to their enormous potential for practical application in pharmaceutical and industrial processes. The production of rare sugars using microorganisms and their enzymes, particularly dehydrogenases, has been the prime focus of many reports (Morimoto et al., 1986; Panagiotou et al., 2002; Watanabe et al., 2005). In this study, we move a step further by introducing a highly efficient AfXDH in the conversion of a cheap polyol, xylitol (∼$5/kg), to an expensive rare sugar, l-xylulose (∼$12,600/g). The product of xylitol conversion by AfXDH was determined to be l-xylulose by HPLC. The product exhibited an indistinguishable retention time from that of authentic l-xylulose (Figure 6). It was confirmed by CD spectrometric methods and compared with d- and l-xylulose standards; finally, it was determined to be l-xylulose. The specific optical rotation of the purified product at 3% in H_2_O at 25°C was +29°, whereas those of standard d- and l-xylulose are −33° and +31°, respectively, suggesting that the product has the l-configuration. However, the conversion yield obtained from 30 mM xylitol using purified AfXDH was only 5.8% in the presence of 5 mM NAD^+^, probably owing to NADH product inhibition (*K*_i, NADH_ = 0.25 mM). To achieve a higher product yield in a sustainable manner, a coupling enzymatic system was generated by introducing SpNOX, an NAD^+^ regeneration enzyme (Figure 6).

AfXDH exhibited a half-life of 15.3 h at 45°C, and SpNOX did not lose any activity below 50°C for up to 6 h. Therefore, both AfXDH and SpNOX can be considered stable for the entire course of the coupling enzymatic reaction (6 h). In consideration of the long lifespan of both AfXDH and SpNOX, the coupling enzymatic reaction also could be reasonably expected to be stable. NAD^+^ used in l-xylulose production was recycled by SpNOX. Using 0.5 mM NAD^+^ as the initial coenzyme, AfXDH catalyzed the oxidation of xylitol, forming l-xylulose; SpNOX catalyzed the reduction of H_2_O_2_ and subsequently regenerated the oxidized coenzyme NAD^+^; this consequently maintained low levels of NADH resulting in the acceleration of xylitol oxidation by AfXDH. The reaction parameters were optimized to obtain the highest yield of xylulose by coupling with SpNOX. The optimal pH was changed from 9.5 to 8. The optimal reaction temperature and reaction time were 30°C and 9 h, respectively. The effect of the ratio of SpNOX to AfXDH on l-xylulose production was also investigated by varying the amount of SpNOX at a fixed concentration of AfXDH (10 U ml^−1^). The optimal ratio of SpNOX and AfXDH for the coupling reaction was 10 (Supplementary Figure S6). Therefore, the detailed conditions for the conversion of xylitol to l-xylulose were as follows: 10 U/ml AfXDH, 100 U/ml SpNOX, 0.5 mM NAD^+^, 0.1 M Tris-glycine buffer (pH 8.0), 25°C. In the presence of 0.5 mM NAD^+^, the yield of l-xylulose obtained from 15 g L^−1^ (100 mM) of xylitol using purified AfXDH without coupling with SpNOX was merely 0.6% (0.09 g L^−1^ of l-xylulose). The yield was remarkably enhanced to 87% [13.1 g L^−1^ of l-xylulose (87 mM)] by coupling AfXDH with SpNOX (Figure 6C). The productivity of 2.18 g L^−1^ h^−1^ was obtained using only 0.5 mM initial NAD^+^. Khan et al. (1991) reported 0.5 g L^−1^ h^−1^ and 80% of l-xylulose productivity and yield, respectively, using resting recombinant cells when *Pantoea ananatis* ATCC 43072 *xdh* gene was overexpressed in *E. coli*. Usvalamp et al. (2009) reported 1.5 g L^−1^ h^−1^ of l-xylulose productivity using *E. coli* BPT228 resting recombinant cells. The productivity of the purified AfXDH was higher than that of the whole-cell systems reported. However, using resting cells for l-xylulose production has several advantages over the use of purified enzymes: regeneration of cofactors, stability of intracellular enzymes, and easy separation of l-xylulose (Usvalampi et al., 2009; Meng et al., 2016; Tesfay et al., 2021). The process using resting recombinant cells overexpressing AfXDH is currently under investigation in our laboratory.

## Conclusion

In summary, we characterized a highly active XDH from *A. flavus* NRR3357. The evidence from biophysical property analyses and bioinformatics studies suggests that AfXDH is a member of the MDR family. The AfXDH enzyme shares common biochemical and biophysical properties with previously characterized XDHs but shows the highest *k*_*cat*_ value and thermostability among all previously reported XDHs. Detailed computational analysis of AfXDH and its kinetic parameters for xylitol as its substrate have been provided. As an application of AfXDH, we produced l-xylulose from xylitol using AfXDH. To the best of our knowledge, this is the first report regarding AfXDH and its coupling with the SpNOX NAD^+^ regeneration system for l-xylulose production. The coupling enzymatic system (AfXDH-SpNOX) achieved a sustainable and efficient production of l-xylulose, yielding this rare sugar’s highest recorded production.

## Data Availability

The datasets presented in this study can be found in online repositories. The names of the repository/repositories and accession number(s) can be found in the article/Supplementary Material.
