# Supplementary material for: Characterization of a xylitol dehydrogenase from Aspergillus flavus and its application in l-xylulose production
Source: Front Bioeng Biotechnol. 2022 Sep 12;10:1001726. doi: 10.3389/fbioe.2022.1001726 (PMC9512048; doi:10.3389/fbioe.2022.1001726)
Supplement: Supplementary file 1 [file DataSheet1.docx]

**Supplementary material for**

Characterization of a xylitol dehydrogenase from *Aspergillus flavus* and its application in l-xylulose production

Anurag Kumar^✝^, Jinglin Li^✝^, Sanath Kondaveeti, Bakul Singh, Ramasamy Shanmugam, Vipin C. Kalia, In-Won Kim, and Jung-Kul Lee*

*Department of Chemical Engineering, Konkuk University, 1 Hwayang-Dong, Gwangjin-Gu, Seoul 05029, Republic of Korea*

*Corresponding author

Postal address: Department of Chemical Engineering, Konkuk University, 1 Hwayang-dong, Gwangjin-gu, Seoul 05029, Republic of Korea

Tel: +82-2-450-3505. Fax: +82-2-458-3504. E-mail: jkrhee@konkuk.ac.kr

**Table of Contents**

|  |  | Page No. |
| --- | --- | --- |
| Materials | Materials | 3 |
| Figure S1. | The expression (A) and purification (B) of the SpNOX at 25 ºC. | 4 |
| Figure S2. | Multiple sequence alignment of AfXDH and ReXDH protein sequences with respective templates. The highly conserved active site residues (SER49, GLU158, and ARG304) of AfXDH are highlighted in a red box. | 5 |
| Figure S3. | Generated model of AfXDH from homology modeling shows protein with NAD^+^ and zinc in the bound state. | 6 |
| Figure S4. | Schematic of the Ramachandran plot for the model showing the integrity of the AfXDH structure. | 7 |
| Figure S5. | Schematic of the Ramachandran plot for the model of the ReXDH structure. | 8 |
| Figure S6. | The effect of the ratio of SpNOX to AfXDH in the coupling reaction. | 9 |
| Table S1. | Hydrogen bonds and distance between xylitol and active site residues of AfXDH and ReXDH. | 10 |

**Materials**

Reagents for the polymerase chain reaction (PCR), Ex Taq DNA polymerase and T4 DNA ligase, were purchased from TaKaRa (TakaraBio, Japan). A genomic DNA extraction kit and the pGEM-T easy vector were purchased from Promega (Madison, USA). Restriction enzymes were obtained from New England Biolabs (MA, USA). The pQE80L expression vector and the plasmid isolation kit were obtained from Novagen (WI, USA); the Ni-NTA Superflow column for purification was purchased from Qiagen (Hilden, Germany). Oligonucleotide primers were obtained from Macrogen Inc. (Seoul, Republic of Korea). Electrophoresis reagents were from Bio-Rad (CA, USA), and all chemicals for the assay were from Sigma Aldrich (St. Louis, MO, USA).


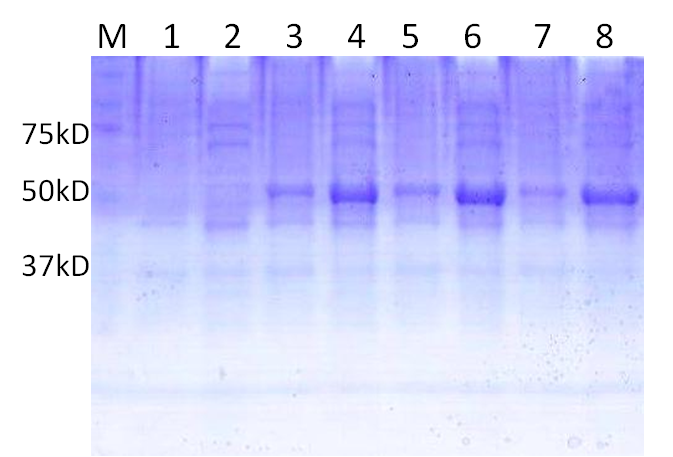

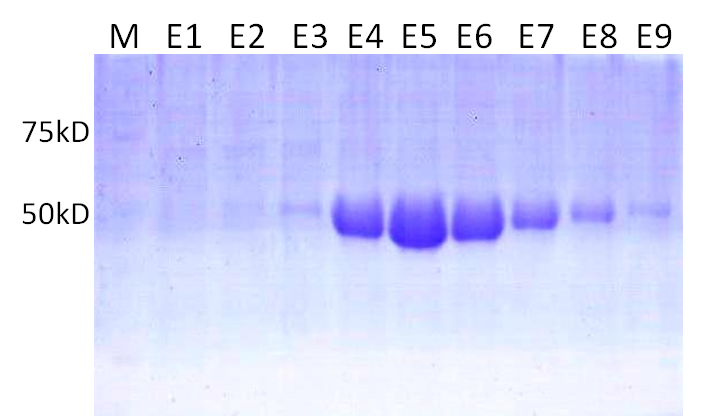


(A) (B)

**Fig. S1.** The expression (A) and purification (B) of the SpNOX at 25 ºC. M= marker, 1= 0 mM IPTG (pellet), 2= 0 mM IPTG (supernatant), 3= 0.05 mM IPTG (pellet), 4= 0.05 IPTG (supernatant), 5= 0.1 mM IPTG (pellet), 6= 0.1 mM IPTG (supernatant), 7= 0.2 mM IPTG (pellet), 8= 0.2 mM IPTG (supernatant), E1-E9= eluted fraction using elution buffer containing 250 mM imidazole.


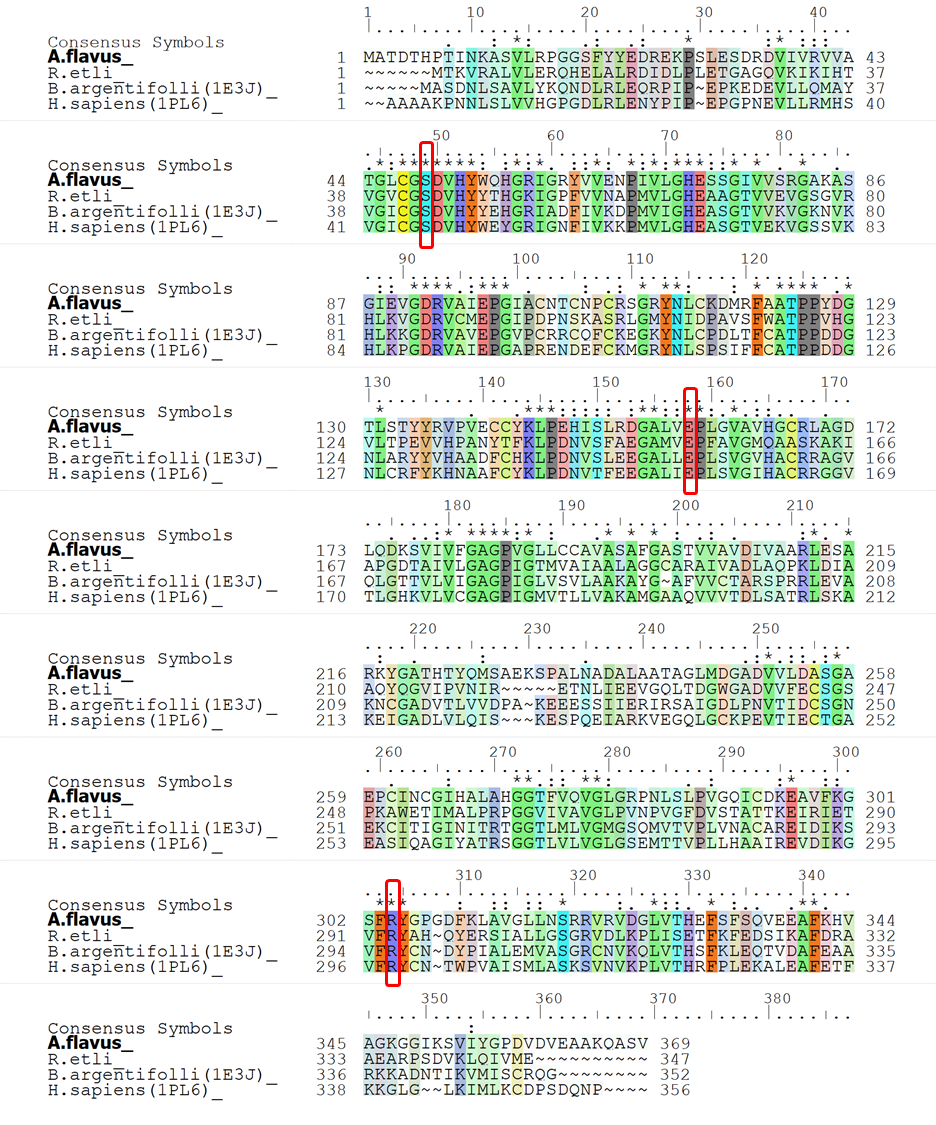


**Fig. S2.** Multiple sequence alignment of AfXDH and ReXDH protein sequences with respective templates. The highly conserved active site residues (SER49, GLU158, and ARG304) of AfXDH are highlighted in a red box. PDB ids of templates are in parenthesis.


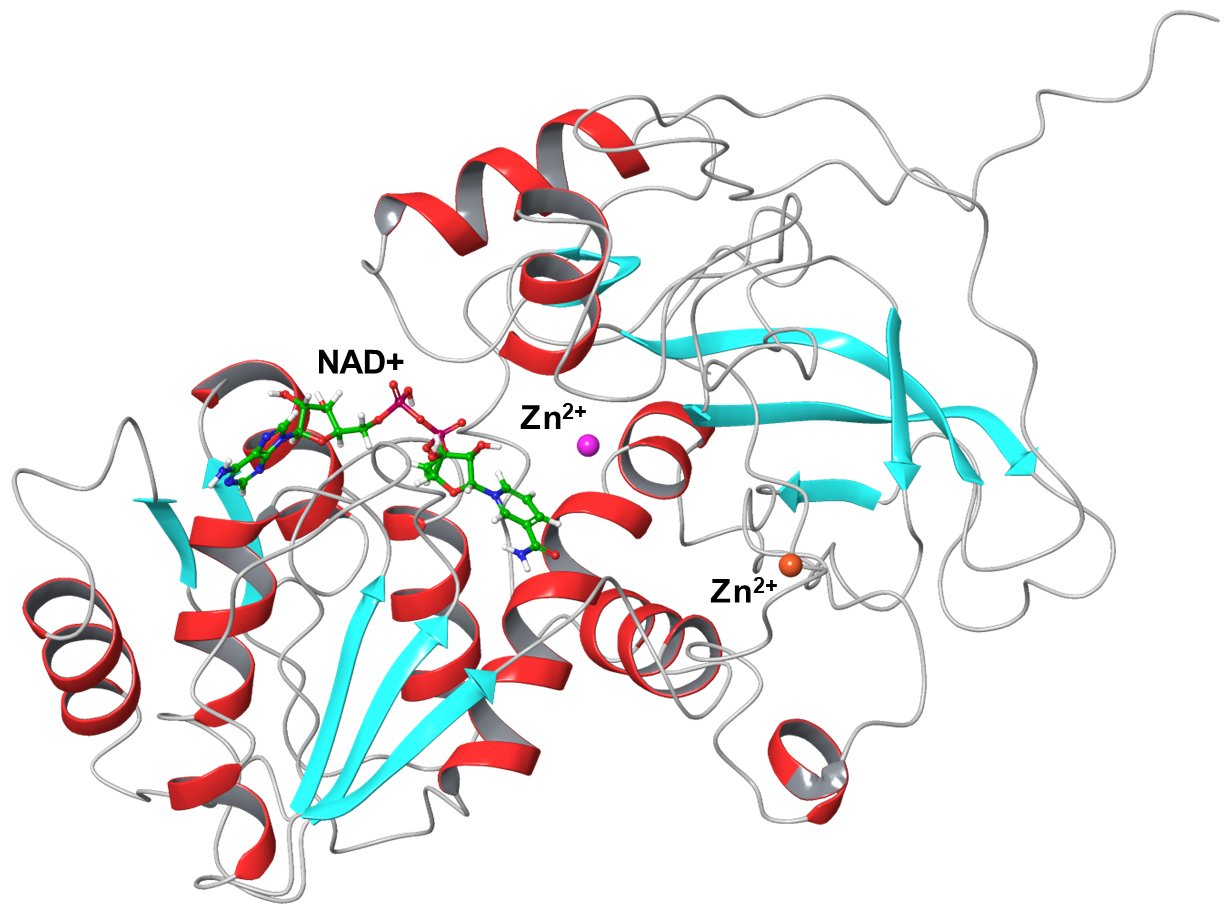


**Fig. S3.** Generated model of AfXDH from homology modeling shows protein with NAD+, catalytic zinc. Protein (cartoon), NAD^+^ (ball and stick), catalytic zinc (magenta sphere), structural zinc (orange sphere).


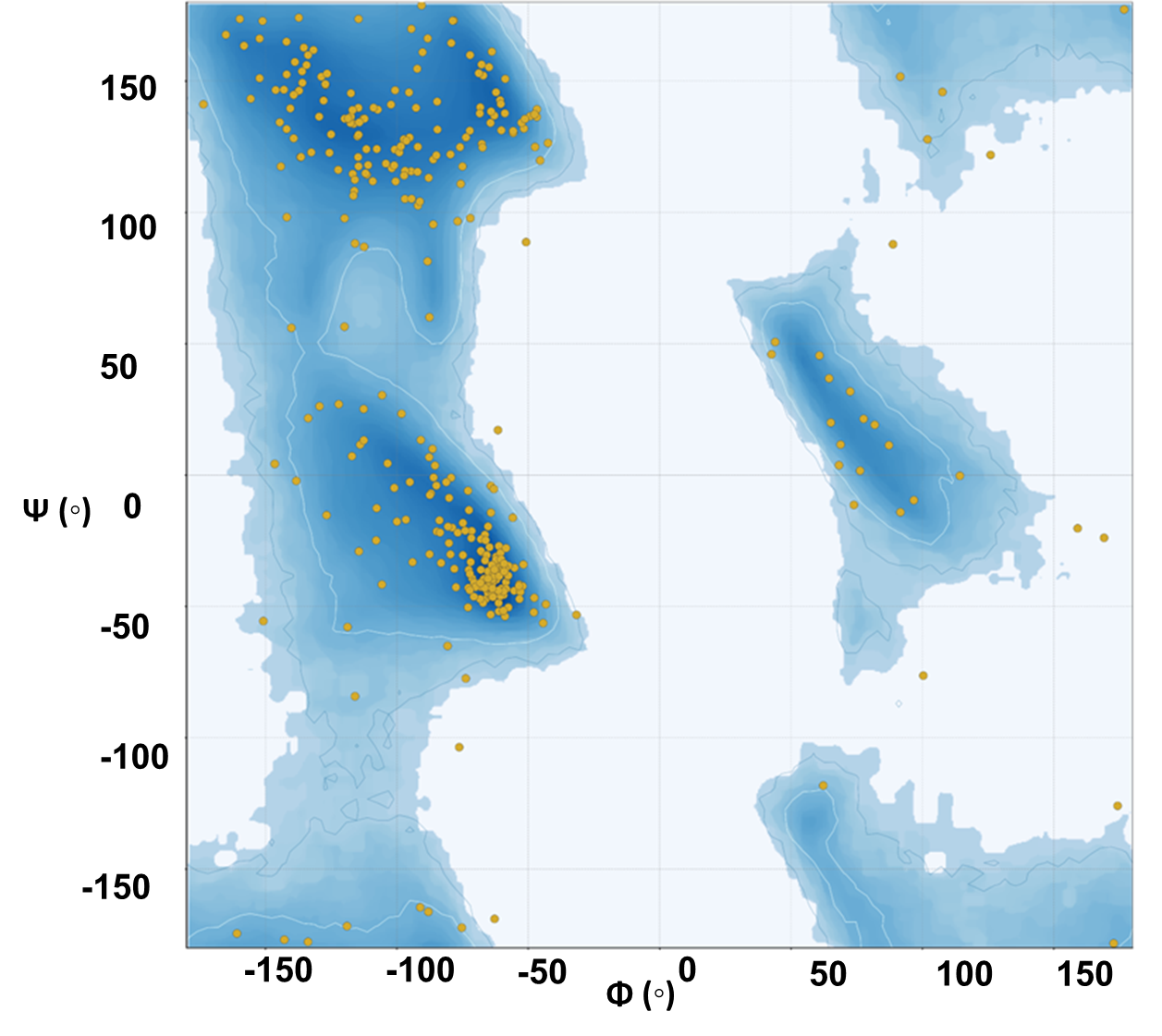


**Fig. S4.** Schematic of the Ramachandran plot for the model showing the integrity of the AfXDH structure. The homology model of AfXDH (Figure S2) was validated by the Ramachandran plot with 95.22% of residues in the favored region, 2.73 % in allowed regions, and 2.04% in disallowed regions. The dark navy blue is the favored region, the light navy blue surrounding the dark navy blue is the allowed region, and the white is the disallowed region. The yellow dots show the residues of the AfXDH.

**
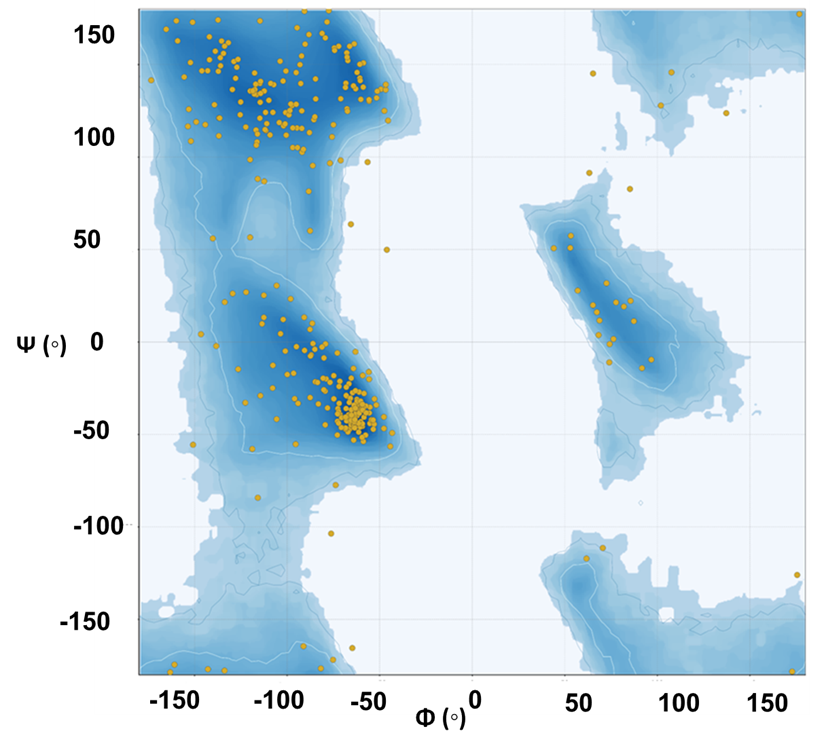
**

**Fig. S5.** Schematic of the Ramachandran plot for the model of the ReXDH structure. The homology model of ReXDH was validated by the Ramachandran plot with 93.45 % of residues in the favored region, 3.49 % in allowed regions, and 1.04 % in disallowed regions. The dark navy blue is the favored region, the light navy blue surrounding the dark navy blue is the allowed region, and the white is the disallowed region. The yellow dots show the residues of the ReXDH.


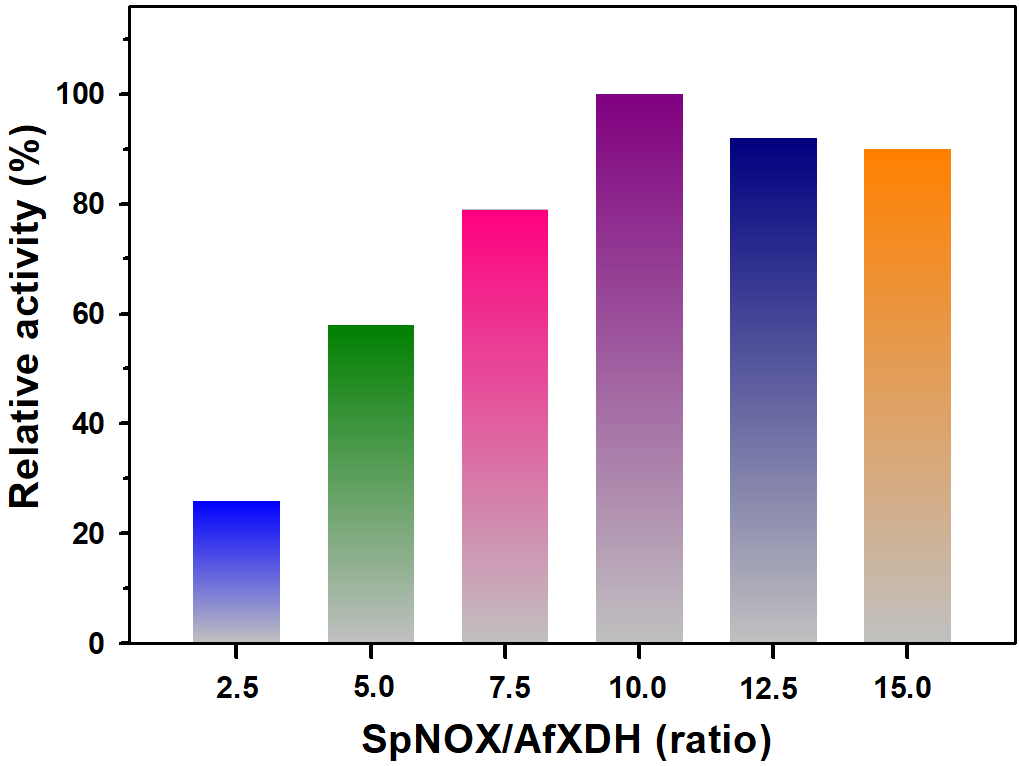


**Fig. S6.** The effect of the ratio of SpNOX to AfXDH in the coupling reaction. A fixed concentration of AfXDH (10 U·mL^-1^) was used.

**Table S1.** Hydrogen bonds and distance between xylitol and active site residues of AfXDH and ReXDH.

| XDH | H-bond donor/atom | H-bond acceptor/atom | Distance (in Å) |
| --- | --- | --- | --- |
| AfXDH | ARG304/NH2 | Xylitol/O5 | 3.5 |
|  | ARG304/NE | Xylitol/O5 | 3.2 |
|  | SER49/OG | Xylitol/O3 | 2.9 |
|  | GLU158/OE1 | Xylitol/O4 | 2.8 |
| ReXDH | GLU152/OE1  SER42/OG | Xylitol/O4  Xylitol/O3 | 2.6  3.2 |
